# Supplementary material for: Pyruvate anaplerosis is a targetable vulnerability in persistent leukaemic stem cells
Source: Nat Commun. 2023 Aug 17;14:4634. doi: 10.1038/s41467-023-40222-z (PMC10435520; doi:10.1038/s41467-023-40222-z)
Supplement: Supplementary file 1 — Supplementary Information [file 41467_2023_40222_MOESM1_ESM.pdf]

Pyruvate Anaplerosis is a Targetable Vulnerability in Persistent Leukaemic Stem Cells

Kevin M. Rattigan[1ɫ], Zuzana Brabcova[1ɫ‡], Daniele Sarnello[1], Martha M. Zarou[1], Kiron Roy[1], Ryan Kwan[2], Lucie de Beauchamp[1], Amy Dawson[1], Angela Ianniciello[1], Ahmed Khalaf[1], Eric R. Kalkman[1], Mary T. Scott[1], Karen Dunn[3], David Sumpton[2], Alison M. Michie[3], Mhairi Copland[3], Saverio Tardito[1,2], Eyal Gottlieb[4], G. Vignir Helgason[1]

[1]Wolfson Wohl Cancer Research Centre; Institute of Cancer Sciences, University of Glasgow, Glasgow, G61 1QH, UK.
[2]Cancer Research UK Beatson Institute, Glasgow G61 1BD, UK.
[3]Paul O'Gorman Leukaemia Research Centre; Institute of Cancer Sciences, University of Glasgow, Glasgow, G12 0ZD, UK.
[4]The Ruth and Bruce Rappaport Faculty of Medicine; Technion-Israel Institute of Technology, Haifa, Israel.

[ɫ]These authors contributed equally

[‡]Deceased

Corresponding author. Vignir.Helgason@Glasgow.ac.uk

Supplementary Information File containing:

Supplementary Figures 1-7

Source Data (in separate files)

Supplementary Tables 1-2

Full Western Blot membranes (uncropped and unprocessed scans of the western blots shown in the supplementary figures and Source Data File)

Supplementary Fig. S1

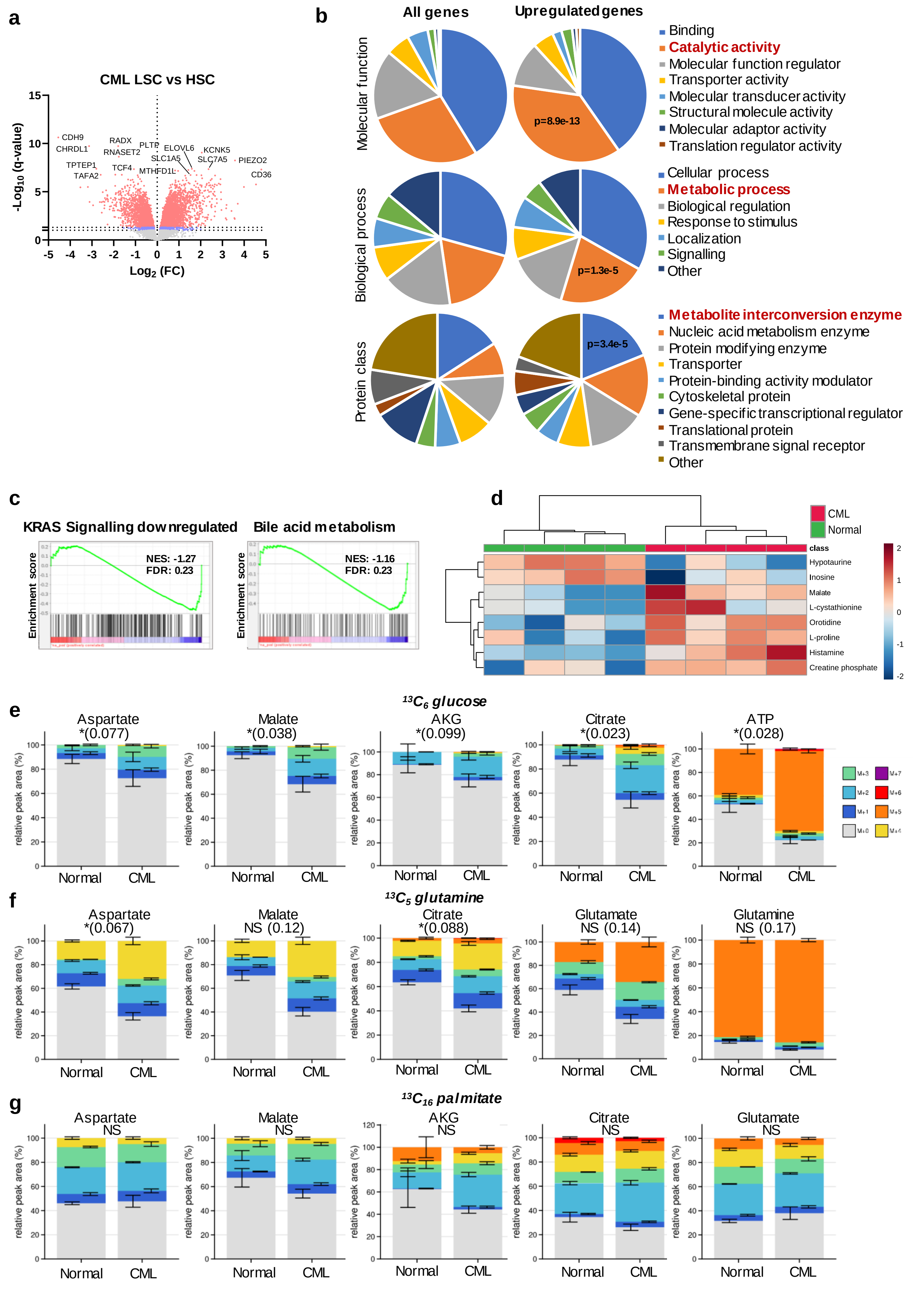

Supplementary Fig. S1. Metabolism is highly deregulated in human CML LSCs (E-MTAB-2581) with LSCs have bias towards glucose metabolism compared to HSCs

(A), Volcano plot of differentially expressed genes. Red coloured genes have q value <0.05 and blue coloured genes have q value <0.1. **(B)**, Results of Panther analysis using indicated sets with result of Fishers exact test (two-sided) shown on pie-chart. **(C)**, Downregulated gene sets in CML. GSEA conducted using Hallmark gene set. **(D)**, Steady state levels of significantly different metabolites ($p<0.1$) in CML CD34+ cells compared to normal CD34+ cells (n=4 each group). Analysis was carried out using MetaboAnalyst 5.0. Multiple t-tests (two-sided) were used, and the Benjamini-Hochberg FDR method was used to correct for multiple comparisons. (Raw data source: Kuntz *et al*, 2017). **(E-G)**, Fractional labelling from $^{13}C_6$ glucose (n=4 normal and patient-derived samples; **(E)**), $^{13}C_5$ glutamine (n=3 normal and n=6 patient-derived samples; **(F)**) and $^{13}C_{16}$ palmitate (n=3 normal and patient-derived samples; **(G)**) into indicated metabolites of CML CD34+ cells and normal CD34+ cells after 24 hours *in vitro* culture. Average and SEM are plotted. For E-G multiple unpaired t-tests (two-sided) were used and the two-stage step-up (Benjamini, Krieger, and Yekutieli) was used to correct for multiple comparisons. Plots were generated using Autoplotter. (LCMS raw data source: Kuntz *et al*, 2017, for **(E)** and **(G)**). Source data are provided as a Source Data file.

Supplementary Fig. S2

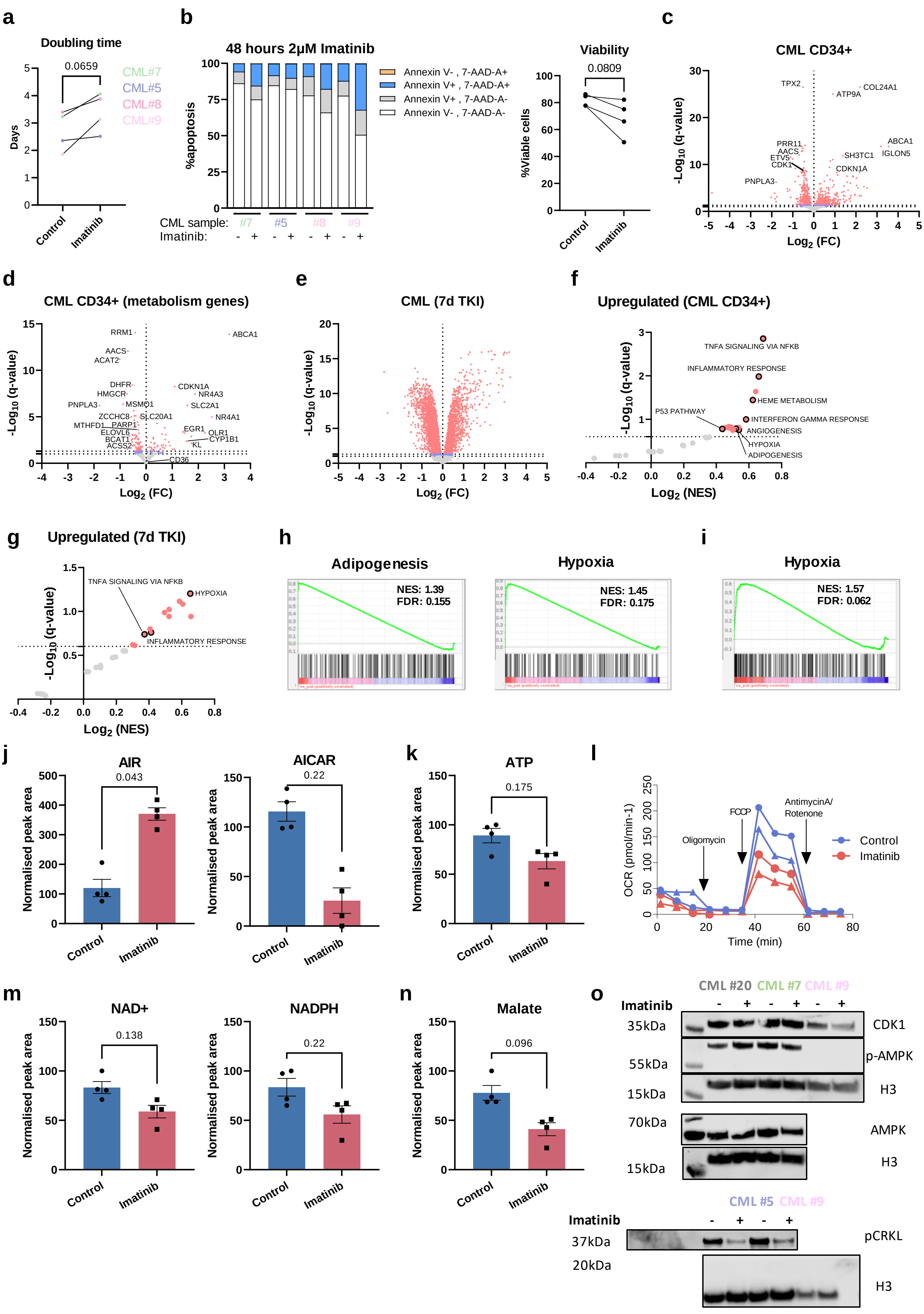

Supplementary Fig. S2. Imatinib partially reverses BCR-ABL driven metabolic reprogramming of human CML LSCs

(A-B), Doubling time **(A)** and viability **(B)** of CML CD34+ cells (n=4 patient-derived samples) treated *in vitro* with 2 μM imatinib for 48 hours. For A and B statistical analysis was performed using a paired t-test (two-sided). **(C-D)**, Volcano plot of differentially expressed genes **(C)** and differentially expressed metabolic genes **(D)**, comparing control and 48 hours imatinib treated CML CD34+ cells (n=4 patient-derived samples). Red coloured genes have q value <0.05 and blue coloured genes have q value <0.1. The Benjamini-Hochberg adjustment was used to correct for multiple comparisons. **(E)**, Volcano plot of differentially expressed genes between control and imatinib treated CML CD34+ cells (E-MTAB-2594) after 28 hours. Red coloured genes have q value <0.05 and blue coloured genes have q value <0.1. The Benjamini-Hochberg adjustment was used to correct for multiple comparisons. **(F)**, GSEA conducted using Hallmark gene sets. The upregulated gene sets are shown. **(G)**, GSEA conducted on E-MTAB-2594 using Hallmark gene sets. The upregulated gene sets are shown. **(H)**, Upregulated gene sets (from **(A)**) shown are adipogenesis and hypoxia. **(I)**, Upregulated gene sets (from **(C)**) shown is hypoxia. **(J, K, M, N)**, Select metabolites from **Fig. 2F** are shown with corresponding corrected p-values shown. Multivariate analysis was carried out using MetaboAnalyst 5.0. The Benjamini-Hochberg FDR method was used to correct for multiple comparisons. Average and SEM are plotted. **(L)** Seahorse profile from CML CD34+ cells pre-treated with 1 μM imatinib for 24 hours (n=2 patient-derived samples, circle denotes CML#19, triangle denotes CML#25). OCR: oxygen consumption rate. **(O)** Western blot with indicated samples are shown. CML CD34+ cells (n=3 patient-derived samples) treated *in vitro* with 2μM imatinib for 24 hours for top 3 samples, 48 hours for bottom two. Top 3 panels are from the same gel, middle 2 panels are both another gel and bottom 2 panels are both from another gel. Source data are provided as a Source Data file.

Supplementary Fig. S3

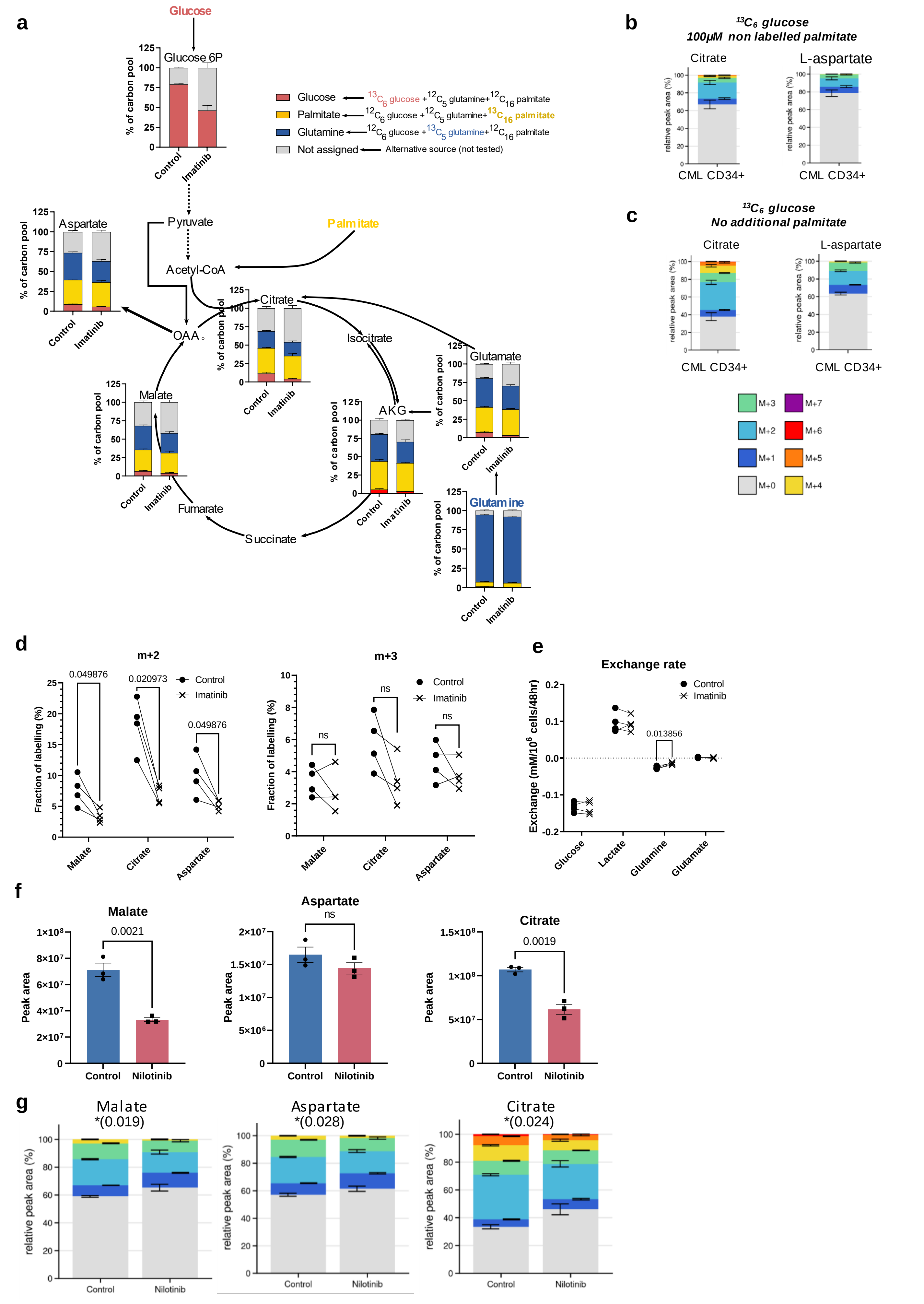

Supplementary Fig. S3. Imatinib disrupts nutrient contribution to TCA cycle in CML CD34+ cells

(A), Fractional labelling (m+2 and above) to carbon pool from $^{13}C_6$ glucose, $^{13}C_5$ glutamine and $^{13}C_{16}$ palmitate in control and imatinib treated CML CD34+ cells (n=4 patient-derived cells per group). Average and SEM are plotted. Cells were treated with 2µM imatinib for 48 hours. **(B-C)**, Fractional labelling from $^{13}C_6$ glucose into indicated metabolites are shown (n=4 patient-derived samples) after 24 **(C)** or 48 **(B)** hours. Medium contained 100 µM non-labelled palmitate **(B)** or no additional non-labelled palmitate **(C)**. Plots were generated using Autoplotter, average and SEM are plotted. **(D)**, Shown are the m+3 and m+2 fractions for indicated metabolites (n=4 patient-derived cells per group). Multiple paired t-tests (two-sided) were used and the two-stage step-up (Benjamini, Krieger, and Yekutieli) was used to correct for multiple comparisons. **(E)**, Exchange rate of indicated extracellular metabolites in control and 2µM imatinib treated CML CD34+ cells (n=4 patient-derived samples) after 48 hours culture with $^{13}C_6$ glucose. Multiple paired t-tests (two-sided) were used and the two-stage step-up (Benjamini, Krieger, and Yekutieli) was used to correct for multiple comparisons. **(F)**, Steady state and **(G)**, fractional labelling (m+2 and above $^{13}C_6$ glucose) in control and nilotinib treated CML CD34+ cells (n=3 patient-derived cells per group). Average and SEM are plotted. Cells were treated with 1µM nilotinib for 24 hours. Unpaired t-tests (two-sided) were used on data from F and G. Source data are provided as a Source Data file.

Supplementary Fig. S4

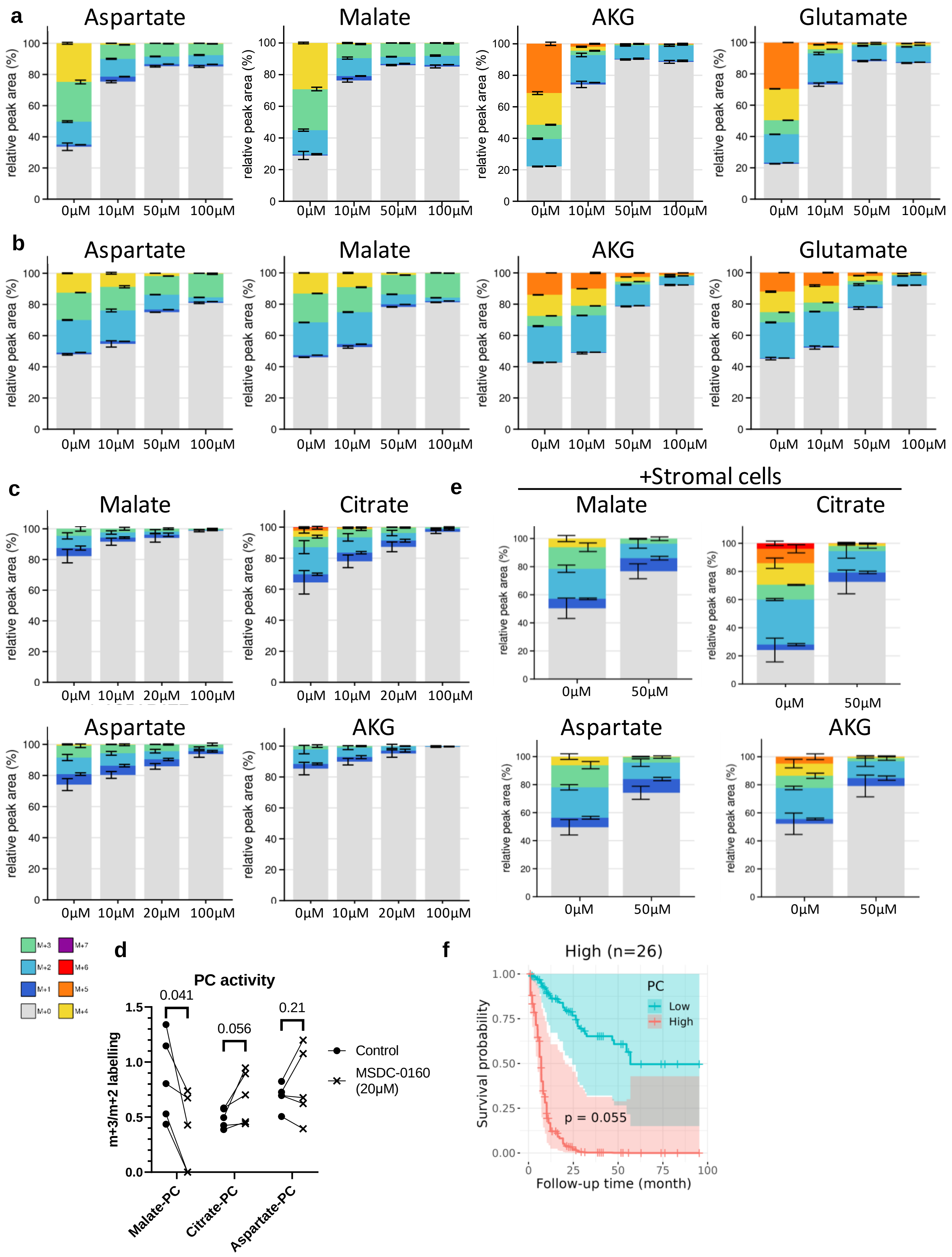

Supplementary Fig. S4. CML CD34+ cells have PC activity that can be abrogated using MPC1/2 inhibitor

(A-B), Fractional labelling from $^{13}C_6$ glucose into indicated metabolites from K562 cells are shown (n=3 independent cultures) after 48 hours (A) or 24 (B) hours culture. Cells were treated with indicated concertation of UK-5099 **(A)** or MSDC-0160 **(B)**. Plots were generated using Autoplotter. Average and SD are plotted. **(C)**, Fractional labelling from $^{13}C_6$ glucose into indicated metabolites from CML CD34+ cells (patient-derived samples; n=6 for 0µM and 10µM, n=5 for 20µM and 50µM) are shown after 24 hours culture. Cells were treated with indicated concertation of MSDC-0160. Plots were generated using Autoplotter. Average and SEM are plotted. **(D),** PC activity in n=4 patient-derived samples from C quantified using ratio of m+2/m+3. Multiple paired t-tests (two-sided) were used and the two-stage step-up (Benjamini, Krieger, and Yekutieli) was used to correct for multiple comparisons (**E**), Fractional labelling from $^{13}C_6$ glucose into indicated metabolites from CML CD34+ cells (n=3 patient-derived samples) are shown after 24 hours culture in presence of stromal cells. Cells were treated with indicated concertation of MSDC-0160. Plots were generated using Autoplotter. Average and SEM are plotted. **(F)**, Survival plots showing effect of *PC* expression on overall survival in patients with indicated risk cytogenetics, stratified by low or high (20th and 80th percentiles) *PC* expression levels. The 95% confidence intervals were represented by the boundaries of mean ± 1.96 * standard deviation, N refers to number of patients. The effect of the interaction term between *PC* and high cytogenetic risk was estimated by Cox proportional hazards model (HR=2.44, CI=[0.9803, 6.074], z=1.917, two-sided p=0.0552). Source data are provided as a Source Data file.

Supplementary Fig. S5

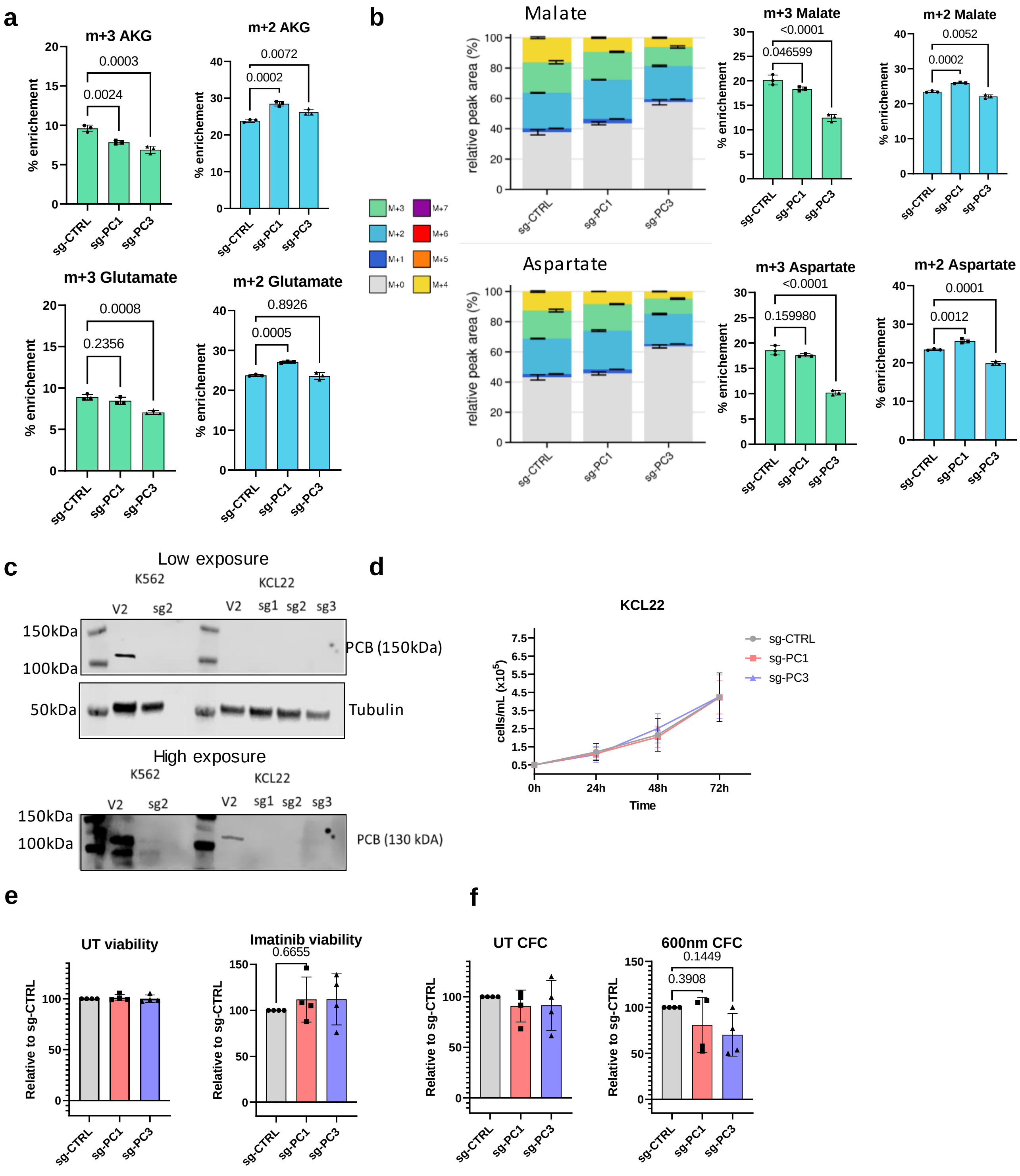

Supplementary Fig. S5 PC ablation or inhibition of mitochondrial pyruvate import fails to sensitises PC-low CML to imatinib

(A), Fractional labelling from $^{13}C_6$ glucose into indicated metabolites of PC knockout cells are shown (n=3 independent cultures) after 24 hours. Plots were generated using Autoplotter, average and SD are plotted. Statistical analyses were conducted on the m+3 or m+2 fraction. **(B)**, **(**Fractional labelling from $^{13}C_6$ glucose into indicated metabolites of PC knockout cells are shown (n=3 independent cultures) after 24 hours. Plots were generated using Autoplotter, average and SD are plotted. Statistical analyses were conducted on the m+3 or m+2 fraction. **(C)**, Western blot of PC and loading control (tubulin) from vector control or PC knockout K562 cells. **(D)**, Proliferation of K562 cells from C. Shown is average and SD from 3 independent experiments. **(E)**, Cells viability was measured using Annexin V and 7AAD. Cells were either left untreated (UT) or exposed for 72 hours to 600nm imatinib or 100nm omacetaxine (OMA). Shown is average and SD from 4 independent experiments. **(F)**, Colony Forming Cell (CFC) potential of control and PK knockout cells. Cells were either left untreated (UT) or exposed for 72 hours to 600nm imatinib. Shown is average and SD from 4 independent experiments. An ordinary one-way ANOVA was used with Dunnett's test used to correct for multiple comparisons for **A, B, E** and **F.** Source data are provided as a Source Data file.

Supplementary Fig. S6

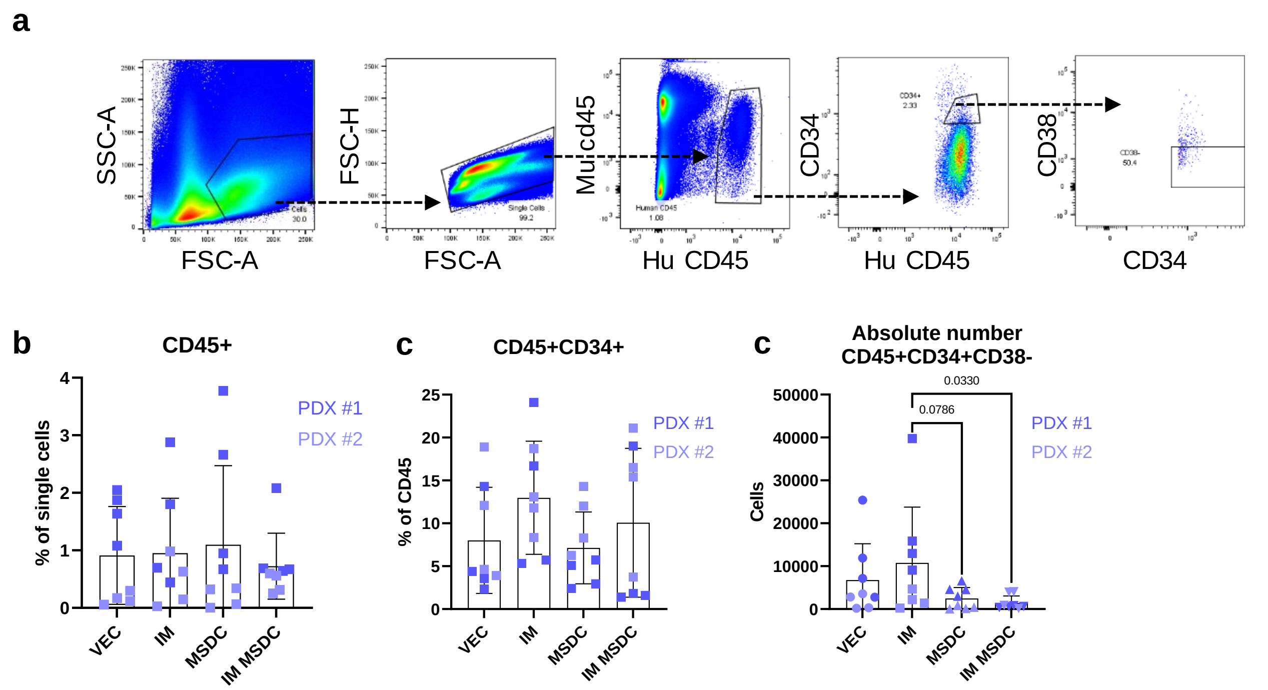

Supplementary Fig. S6. Inhibition of mitochondrial pyruvate transport targets human CML LSCs *in vivo*

(A), Gating strategy is shown for flow cytometry analysis. **(B-C)**, The % of CD45+ cells (from single cells; **(B)**), The % of CD34+ cells (from CD45+; **(C)**), or the absolute number of CD45+CD34+CD38- cells; **(D)** is shown. Average and SD are plotted (n=4 mice, two independent PDX experiments). A Kruskal-Wallis test was used to analyse data. Source data are provided as a Source Data file.

Supplementary Fig. S7

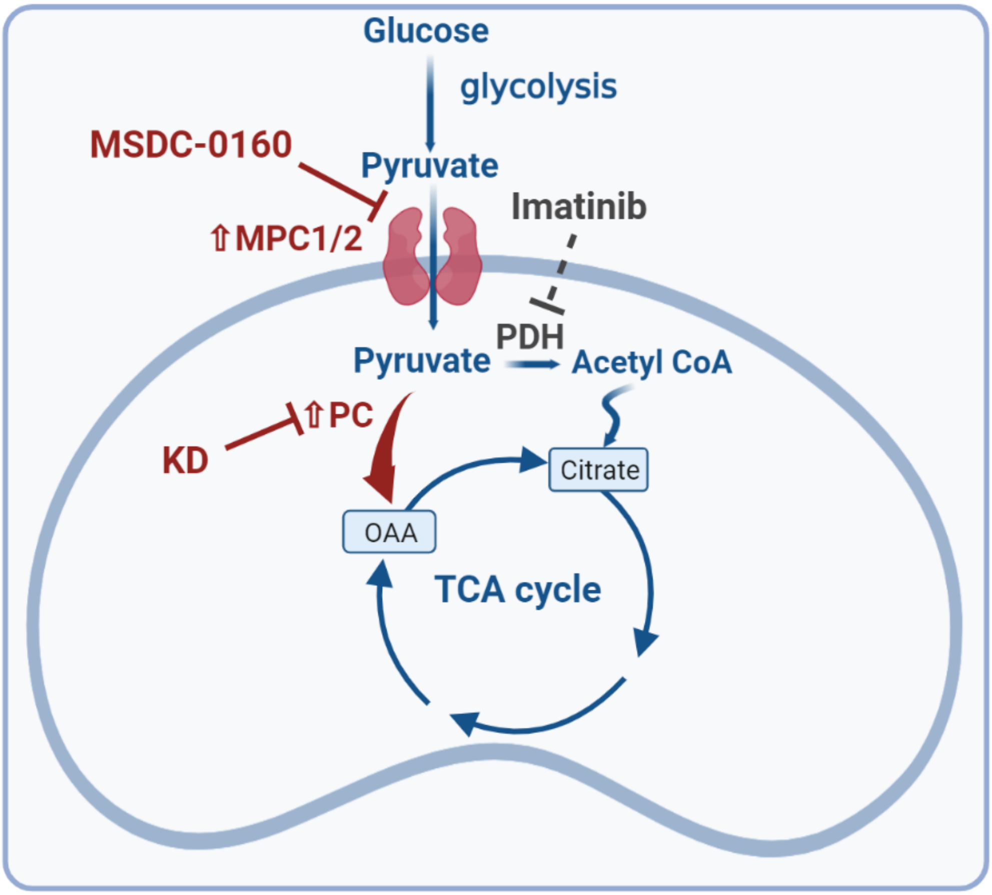

Supplementary Fig. S7. A proposed working model of upregulated pyruvate anaplerosis in CML LSCs.

Metabolism of glucose contributes to upregulated central carbon metabolism in treatment naive CML LSCs. Imatinib partially reverses this except for upregulated MPC1/2 and PC. MSDC-0160 blocks pyruvate imports into mitochondria hence blocking residual glucose oxidation, phenocopying the effects of genetic PC ablation. Created with BioRender.com. (Agreement number: NE25IF9JJP).

SAMPLE	CLINICAL INFORMATION	FIGURES
CML#1	ELN warning in CP on imatinib.	1h-i, 1j, E.1f-g
CML#2	ELN failure Nilotinib at diagnosis -> dasatinib.	1h-i, 1j, E.1f-g
CML#3	ELN failure.	1h, 1j, E.1f, E.3c, 4d-e, E.4c-d, 5f (20µM MSDC-1060)
CML#4	ELN failure Imatinib->nilotinib->dasatinib->SCT.	1h, 1j, E.1f, E.3c
CML#5	ELN failure: BCR-ABL 0.11% at 12 months, MMR by 18 months: not resistant.	1h, 1j, 1k, E.1f, 2b, 2d, 2f-l, E.2a-d, E.2f, E.2h, E.2j-k, E2m-n, E. 2o, 3b-g, E.3a-g, 4g, E.4e
CML#6	Failed imatinib -> dasatinib.	1h, 1j, E.1f, E.3c
CML#7	Optimal response to imatinib: BCR-ABL 0.04% at 12 months.	2b, 2d, 2f-l, E.2a-d, E.2f, E.2h, E.2j-k, E2m-n, E. 2o, 3b-g, E.3a-b, E.3d-g, 4g, E.4e
CML#8	Initial optimal response to imatinib, loss of response -> dasatinib; mutation screen negative; in MMR; optimal response.	2b, 2d, 2f-l, E.2a-d, E.2f, E2h, E.2j-k, E2m-n, 3b-f, E.3a-b, E.3d-e
CML#9	Optimal response to imatinib (MR4).	2b, 2d, 2f-l, E.2a-d, E.2f, E.2h, E.2j-k, E2m-n, E. 2O, 3b-f, E.3a-b, E.3d-e, 4d-e, E.4c-d, 5f (20µM MSDC-1060)
CML#10	Suboptimal response to imatinib 400mg, increased to 600mg, then reduced to 400mg, optimal response: BCR-ABL 0.01%.	4d-e, E.4c-d, 5f (20µM MSDC-1060)
CML#11	ELN warning on dasatinib.	4d-e, E.4c-d, 5f (20µM MSDC-1060)
CML#12	Failed imatinib (compliance issues).	4d-e, E.4c-d
CML#13	ELN warning.	4d-e, E.4c, E.4d, 5f (20µM MSDC-1060)
CML#14	Failed imatinib -> dasatinib, died.	1k, 5f (both doses), 6b-c, E.6a-d
CML#15	No TKI, no molecular monitoring; allogeneic SCT. Cured.	5f (50µM MSDC-1060)
CML#16	Imatinib 400mg; only achieved MR2, treatment failure, continued imatinib due to co-morbidities.	5f (50µM MSDC-1060)
CML#17	Optimal response to imatinib at 3 months: BCR-ABL 0.07%, -> dasatinib due to side effects.	5f (50µM MSDC-1060)
CML#18	ELN warning.	5f (50µM MSDC-1060)
CML#19	Non-optimal response to imatinib 400mg, therapy interrupted due to neutropenia intermittently -> dasatinib 100mg daily: BCR-ABL PCR < 0.87% at 6 weeks.	E.2l, 5f (50µM MSDC-1060)
CML#20	ELN warning, responded to imatinib, achieved MMR on dasatinib	E.2o, 3g, 4g, E.4e
CML#21		1i, E.1g
CML#22	ELN warning, non-optimal response to imatinib, achieved MMR on nilotinib	1g, 1j, 1k, E.1d-e
CML#23	Optimal response to imatinib, MMR at 6 months	1g, 1j, E.1d-e
CML#24	Achieved MMR on imatinib	1g, 1j, E.1d-e
CML#25		E.2l

Supplementary Table 1: Patient samples (at diagnosis) used in this study.

-> refers to next treatment; ELN: European LeukaemiaNet (recommendations for the management of CML); E: Extended Data Figure; CP: chronic phase; SCT: stem cell transplantation; MMR: major molecular response; MR4: BCR-ABL ≤0.01% international scale (IS); MR2: BCR-ABL <1% IS. All samples were >95% positive for BCR-ABL by FISH.

Supplement	Supplier	Catalogue number	Final Concentration
**Albumax II**	Thermofisher Scientific	11021037	1mg/mL
**Human insulin**	Merck	I9278-5ML	10µg/mL
**Glutamine**	Thermofisher Scientific	25030-024	0.65mM
**$^{13}UC_5$ Glutamine**	Cambridge Isotopes	CLM-1166-0.25	0.65mM
**Glucose**	Merck	G7021-100G	5.5mM
**$^{13}UC_6$ Glucose**	Cambridge Isotopes	CLM-1396	5.5mM
**Sodium Palmitate**	Merck	P9767	100uM
**$^{13}UC_{16}$ Sodium Palmitate**	Cambridge Isotopes	CLM-6059	100uM
**Transferrin**	Merck	T4132-100MG	7.5µg/mL
**Beta mercaptoethanol**	Merck	21980	100µM
**Pyruvate**	Merck	S8636-100ML	100µM
**P/S**	Thermofisher Scientific	15140122	1%
**SCF**	Peprotech	3000-07	0.20ng/mL
**G-CSF**	Peprotech	300-23	1 ng/mL
**GM-CSF**	Peprotech	300-03	0.20ng/mL
**IL6**	Peprotech	200-06	1 ng/mL
**MIP-a**	Peprotech	300-08	0.20ng/mL
**LIF**	Peprotech	300-05	0.05ng/mL

Supplementary Table 2: Supplements added for medium formulation used in this study

S2o top panel

Order (L-R): Ladder, CML #20, CML #20+imatinib, CML#7, CML#7+imatinib, CML#9, CML9+imatinib

CDK1

35kDA

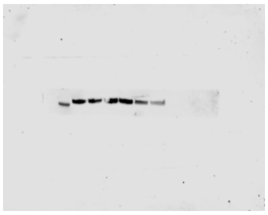

pAMPK

70kDA

55kDA

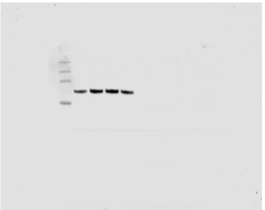

H3

15kDA

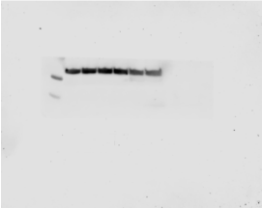

S2o middle panel

Order (L-R): Ladder, CML #20, CML #20+imatinib, CML#7, CML#7+imatinib

AMPK

70kDA

55kDA

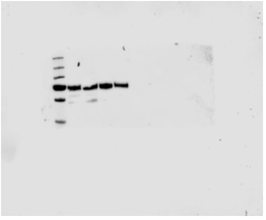

H3

15 kDA

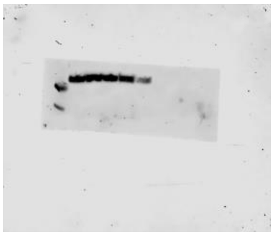

S2o bottom panel

Order (L-R): Ladder, CML #5, CML #5+imatinib, CML#9, CML#9+imatinib, (lanes 1-2 were cml sample with low and unequal protein, 7,8 are normal samples), lane 9 is ladder and lane 10 and 11 are K562 and KCL22

pCRKL

55kDa

37kDa

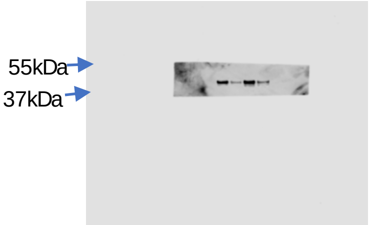

H3

20kDa

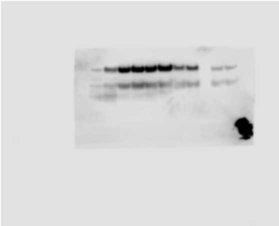

5a

PCB (130 kDA)

Order (L-R): Ladder, V2, g1, g3

150 kDA

100 kDA

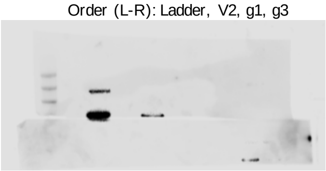

Tubulin

Order (L-R): Ladder, V2, g1, g3

50 kDA

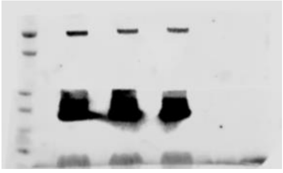

S5c

PCB (130 kDA) low exposure

Order (L-R): Ladder, K562 (V2, g2), Ladder, KCL22 (V2, g1, g2, g3, g4)

150 kDA

100 kDA

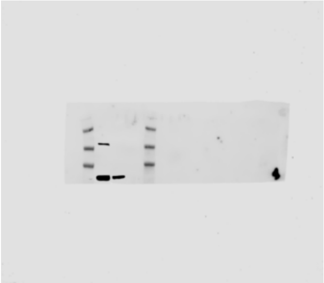

PCB (130 kDA) high exposure

Order (L-R): Ladder, K562 (V2, g2), Ladder, KCL22 (V2, g1, g2, g3, g4)

150 kDA

100 kDA

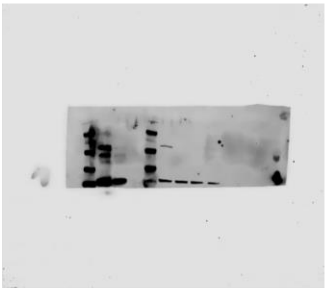

Tubulin

Order (L-R): Ladder, K562 (V2, g2), Ladder, KCL22 (V2, g1, g2, g3, g4)

50 kDA

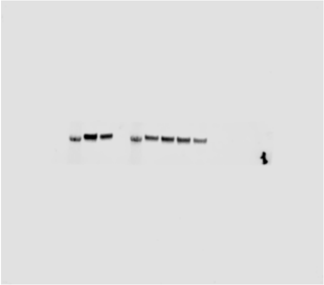

Full Western Blot membranes

Full Western Blot membranes

Full membranes of western blots used either as panels in figures (as indicated)
