# Supplementary material for: Pyruvate anaplerosis is a targetable vulnerability in persistent leukaemic stem cells
Source: Nat Commun. 2023 Aug 17;14:4634. doi: 10.1038/s41467-023-40222-z (PMC10435520; doi:10.1038/s41467-023-40222-z)
Supplement: Supplementary file 2 — Reporting Summary [file 41467_2023_40222_MOESM2_ESM.pdf]

nature portfolio

Corresponding author(s): G. Vignir Helgason

Last updated by author(s): 21/06/2023

Reporting Summary

Nature Portfolio wishes to improve the reproducibility of the work that we publish. This form provides structure for consistency and transparency in reporting. For further information on Nature Portfolio policies, see our Editorial Policies and the Editorial Policy Checklist.

Statistics

For all statistical analyses, confirm that the following items are present in the figure legend, table legend, main text, or Methods section.

n/a	Confirmed	
☐	☒	The exact sample size (*n*) for each experimental group/condition, given as a discrete number and unit of measurement
☐	☒	A statement on whether measurements were taken from distinct samples or whether the same sample was measured repeatedly
☐	☒	The statistical test(s) used AND whether they are one- or two-sided<br>*Only common tests should be described solely by name; describe more complex techniques in the Methods section.*
☐	☒	A description of all covariates tested
☐	☒	A description of any assumptions or corrections, such as tests of normality and adjustment for multiple comparisons
☐	☒	A full description of the statistical parameters including central tendency (e.g. means) or other basic estimates (e.g. regression coefficient) AND variation (e.g. standard deviation) or associated estimates of uncertainty (e.g. confidence intervals)
☐	☒	For null hypothesis testing, the test statistic (e.g. *F*, *t*, *r*) with confidence intervals, effect sizes, degrees of freedom and *P* value noted<br>*Give P values as exact values whenever suitable.*
☒	☐	For Bayesian analysis, information on the choice of priors and Markov chain Monte Carlo settings
☐	☒	For hierarchical and complex designs, identification of the appropriate level for tests and full reporting of outcomes
☒	☐	Estimates of effect sizes (e.g. Cohen's *d*, Pearson's *r*), indicating how they were calculated

Our web collection on statistics for biologists contains articles on many of the points above.

Software and code

Policy information about availability of computer code

Data collection	For flow cytometry, stained cells were collected by FACSVerseTM Flow Cytometer or BDAria Fusion Flow Cytometer (BD Biosciences, BD FACSuite version 1.0.6.5320). For mass spectrometry, spectra were acquired using a qExactive Plus Orbitrap Mass Spectrometer (Thermo Fisher Scientific) operating in polarity switching mode. The Mass Spectrometer was coupled online to LC system composed of a ZIC-pHILIC column (SeQuant, 150 × 2.1mm, 5µm, Merck KGaA) with a ZIC-pHILIC guard column (SeQuant, 20 × 2.1mm) with an UltiMate 3000 HPLC system (Thermo Fisher Scientific). For RNA-seq, libraries were generated with the TruSeq Stranded mRNA LT Kit (Illumina) and ran on the Illumina Next-Seq 500 using the Illumina High-Output 75 cycles kit (2 × 36 cycles, paired end reads, single index). FastQ files were prepared with bcl2fastq (v. 2.20.0.422, Illumina).
Data analysis	BD FACSuite version (BD FACSVerseTM Flow Cytometer, BD FACSuite version 1.0.6.5320) or FACSDiva version (BD FACSAria Fusion, BD FACSDiva Software v8.0.1). Flow cytometry data was analyzed using FlowJo version 10Flow cytometry data was analyzed using FlowJo version 10.<br><br>Data acquisition was achieved with Thermo Xcalibur 4.3.73.11 software. The mass spectrometry files were analysed using using Tracefinder 4.1 software (Thermo Fisher Scientific). Here the peak areas of different metabolites were determined with metabolites identified by accurate mass of the singly charged ion and by known retention times on the pHILIC column. A commercially available standard compound mix (Merck: MSMLS-1EA) had been analysed previously on our LC-MS system to determine accurate ion masses and retention times. The 13C labelling was determined by quantifying peak areas for the accurate mass of all isotopologues of each metabolite and Autoplotter (version 2.3D) was used to correct for natural abundance.<br><br>For RNA-seq data, QC, alignment, and parsing of files into count matrices was performed in command line, with subsequent differential gene expression (DGE) analysis performed in R (1.2.1335). Adapter trimming was performed using Scythe (version 0.981), and Sickle (version

0.940), was used to trim bases with quality scores of less than 20. Prior to and after this pre-processing fastqc (version 0.11.2) was run to ascertain sequence quality, alongside the efficacy of the pre-processing steps. Trimmed reads were indexed and aligned using Hisat2 (version 2.1.0). Hisat2 indexes (GRCh38 genome_tran) were obtained from the John Hopkins Center for Computational Biology, 2020. Samtools (version 0.1.19044428cd) view was used to convert the resulting .sam to .bam files, whilst samtools sort was used to sort the .bam files. Assembly was achieved through the use of stringtie (John Hopkins Center for Computational Biology, 2020), with output .gtf files converted to count matrices using the python script prepDE.py (stringtie version 1.3.3b.Linux_x86_64). Reads were assembled using an annotated reference human genome (GRCh38.p13), obtained from GENCODE (GENECODE, 2020). DESeq2 (version 1.26.0) was used to generate results sets from the gene and transcript count matrices. G genes with read counts too low to allow for the calculation of p and adjusted p-values (padj: Benjamini-Hochberg) were removed from the data sets leaving gene and transcript counts of sizes 16,069 and 45,218 respectively. Microarray datasets were analysed using Limma (version 3.34.9)

GSEA (version 4.1) was conducted on pre-ranked lists (ranked by pi score calculated by multiplying LOG fold change by -LOG (corrected p-value)).

Statistical analysis was performed using R Studio version 1.1.4., MetabonAnalyst 5.0, or Graphpad Prism 9.1. LC-MS peak intensities were R-log-transformed and mean-centered to ensure normality of data. For in vitro and in vivo data, normality was tested with non-parametric tests being used on non-parametric data.A multivariable Cox proportional hazards model was fitted to TCGA data in R software. Two datasets were analysed, one including and the other excluding the FAB M3 subtype.

To simplify the model, a backward stepwise model selection procedure was applied to the complex Cox survival model, which originally included age, sex, FLT3_ITD, protocol, transplant_type, PC, FAB, and cytogenetic_risk predictors (full model). The reduced model (reduced model) was obtained by retaining age, FLT3_ITD, protocol, transplant_type, PC, and cytogenetic_risk predictors, while dropping the interaction term between PC and FAB, from the original model.

The resulting models can be represented as:

Full model: proportional hazard ~ age + sex + FLT3_ITD + protocol + transplant_type + PC * (FAB + cytogenetic_risk)

Reduced model: proportional hazard ~ age + FLT3_ITD + protocol + transplant_type + PC * cytogenetic_risk

Survival plot was generated to illustrate the effect of PC expression on overall survival in patients with high-risk cytogenetics, stratified by low or high (20th and 80th percentiles) PC expression levels. The 95% confidence intervals were represented by the boundaries of mean ± 1.96 * standard deviation.

For manuscripts utilizing custom algorithms or software that are central to the research but not yet described in published literature, software must be made available to editors and reviewers. We strongly encourage code deposition in a community repository (e.g. GitHub). See the Nature Portfolio guidelines for submitting code & software for further information.

Data

Policy information about availability of data

All manuscripts must include a data availability statement. This statement should provide the following information, where applicable:

- Accession codes, unique identifiers, or web links for publicly available datasets
- A description of any restrictions on data availability
- For clinical datasets or third party data, please ensure that the statement adheres to our policy

The expression profiling RNA-seq data generated in this study have been deposited in public Gene Expression Omnibus (GEO) database under accession code GSE216837 [https://www.ncbi.nlm.nih.gov/geo/query/acc.cgi?acc=GSE216837].
The publicly available datasets used in this study are available in the EMBL-EBI database under accession code E-MTAB-2581 [https://www.ebi.ac.uk/biostudies/arrayexpress/studies/E-MTAB-2581].
The remaining data are available within the Article, Supplementary Information or Source Data file.

Standard software packages

LCMS data for analysis of patient samples is in source files. Raw LCMS files generated in this study are available upon request to the corresponding author immediately upon approval of biobanks ethical approval panel and access will not be time-limited. The LCMS samples will be maintained long-term (> 10 years) and raw LCMS files will be maintained indefinitely (>10 years on institutes network drive, Redundant Array of Independent Disks (RAID)). Additional information concerning human samples can be obtained from the corresponding author. Source data are provided with this paper.

Research involving human participants, their data, or biological material

Policy information about studies with human participants or human data. See also policy information about sex, gender (identity/presentation), and sexual orientation and race, ethnicity and racism.

Reporting on sex and gender	These results are not sex specific. While more male patient samples were used (17) than female (8) this was due to the amount of cells available for each patient in biobank.
Reporting on race, ethnicity, or other socially relevant groupings	This data was not collected.

Population characteristics	Limited data on follow-up/current status was obtained with the exception being treatment failure
Recruitment	Samples were obtained with ethical approval from Bio-bank. The authors had no role in original collection of samples.
Ethics oversight	All patients gave written informed consent in agreement with the Declaration of Helsinki and with the approval of the National Health Service (NHS) Greater Glasgow and Clyde Institutional Review Board. Ethical approval was granted to the research tissue bank (REC 15/WS/0077) and for using surplus human tissue in research (REC 10/S0704/60).

Note that full information on the approval of the study protocol must also be provided in the manuscript.

Field-specific reporting

Please select the one below that is the best fit for your research. If you are not sure, read the appropriate sections before making your selection.

- [x] Life sciences
- [ ] Behavioural & social sciences
- [ ] Ecological, evolutionary & environmental sciences

For a reference copy of the document with all sections, see nature.com/documents/nr-reporting-summary-flat.pdf

Life sciences study design

All studies must disclose on these points even when the disclosure is negative.

Sample size	No power calculation was performed for the primary or cell line in vitro experiments. Number of independent experiments or number of biological replicated required was determined based on previous experience (Kuntz et al, Nature Medicine volume 23, pages 1234–1240 (2017)) to ensure sufficient data-points without using excessive numbers of patient samples. For in vivo experiment, prior power calculations were used to estimate the number of mice per experimental arm, factoring in the efficacy of treatment observed from the in vitro data.
Data exclusions	No relevant data was excluded in the analysis
Replication	The number of replicates performed is provided manuscript,. This was 3-4 independent replicates for cell line experiments with exact numbers provided in figure legends. Separate patient samples are counted as biological replicates. All experiments for the data and source data is presented, and irrespective of statistical differences be groups, were repeated independently as stated in manuscript.
Randomization	All experimental mice were randomized (by cage) to the various experimental cohorts prior to treatment. LCMS samples were randomized during data acquisition. No other randomization was performed
Blinding	Researchers were not blinded as it was essential to know which treatments were required for each arm of experiments in case of adverse reaction to single agents or combination treatment or it was not possible due to analysis (e.g. CFCs needed to be counted from 6 well plate).

Reporting for specific materials, systems and methods

We require information from authors about some types of materials, experimental systems and methods used in many studies. Here, indicate whether each material, system or method listed is relevant to your study. If you are not sure if a list item applies to your research, read the appropriate section before selecting a response.

Materials & experimental systems

n/a	Involved in the study	
[ ]	[x]	Antibodies
[ ]	[x]	Eukaryotic cell lines
[x]	[ ]	Palaeontology and archaeology
[ ]	[x]	Animals and other organisms
[ ]	[x]	Clinical data
[x]	[ ]	Dual use research of concern
[x]	[ ]	Plants

Methods

n/a	Involved in the study	
[x]	[ ]	ChIP-seq
[ ]	[x]	Flow cytometry
[x]	[ ]	MRI-based neuroimaging

Antibodies

Antibodies used	For apoptosis and CD34 analysis, cells were stained with Annexin V (fluorescein isothiocyanate (FITC, BioLegend: Cat# 640906, 5 uL/test), 7-AAD (BD Bioscience: Cat# 559925, 5uL/test) and CD34+ (APC, BD Bioscience, clone 581, Cat# 555824, 2uL/test) in 50uL Hanks' Balanced Salt Solution (HBSS) for 20 minutes (room temperature in dark).<br><br>For PDX experiments, cells were stained (300µl/test) with anti-mouse (APC-Cy7 BD Biosciences, clone 30-F11, Cat# 557659, RRID: AB_396774, 1 µl), anti-human CD45 (FITC; BD Biosciences, clone HI30, Cat# 555482, RRID: AB_395874, 10 µl), anti-human CD34 (APC; BD Biosciences, clone 581, Cat# 555824, RRID: AB_398614, 2 µl) and anti-human CD38 (PerCP; BioLegend, clone HIT2, Cat# 303520, RRID: AB_893313, 2 µl) antibodies for 20 minutes in dark (room temperature) prior to flow cytometry analysis.

	For western blotting, primary antibodies used for overnight incubation (4C) were p-AMPK (Cell signalling, Cat #2531, diluted 1:1,000), AMPK (Cell signalling, Cat #2532, diluted 1:1,000), p-CRKL (Cell signalling, Cat #3181, diluted 1:500), CDK (Cell signalling, clone POH1, Cat #9116, diluted 1:1000), PC (Proteintech Cat# 16588-1-AP) and H3 (Active Motif, clone MABI 0301, Cat #39763 diluted 1:1,400). The membranes were rinsed three times with TBS-T, then incubated with secondary HRP-linked antibodies; Anti-rabbit IgG HRP-linked Ab (Cell Signalling Cat#7074, diluted 1:10,000) and Anti-mouse IgG HRP-linked Ab (Cell Signalling Cat#7076, diluted 1:10,000), for 1 hour at room temperature.
Validation	AMPK: validated in various cell lines by manufacturer<br>p-AMPK: validated by manufacturer using AICAR treatment or starvation to induce increase<br>p-CRKL: validated by manufacturer using phosphatase treated K562 cell lysate and here using imatinib on CMLsamples (Figure S2O)<br>CDK: validated by manufacturer using siRNA knock down combined with immunofluroscent analysis.<br>h3: validated by manufacturer using sodium butyrate (inhibits deacetlyation).<br>PC: validated by manufacturer (IP) as well as knock out generated in this study (Figure 5A and Figure S5C).<br>annexin V-FITC: validated in cell lines by manufacturer as reactive to all mammalian species and authors using complementary measurement of cell death (7-AAD)<br>muCD45-APC-CY7: validated by manufacturer on mouse splenic leukocytes alongside isotype control<br>huCD45-FITC: validated by manufacturer on human peripheral blood lymphocytes alongside isotype control<br>huCD34-APC: validated by manufacturer on human peripheral blood stem cells<br>huCD38-PerCP: Validated by manufacturer on human peripheral blood lymphocytes alongside isotype control

Eukaryotic cell lines

Policy information about cell lines and Sex and Gender in Research

Cell line source(s)	K562, KCL22 and HEK-293FT cell lines were originally purchased from DSMZ and cultured following the manufacturer's instructions.
Authentication	K562, KCL22 and HEK-293FT cell lines were authenticated using short-tandem repeat (STR) profiling
Mycoplasma contamination	K562, KCL22 and HEK-293FT cell lines were tested negative in-house for mycoplasma contamination
Commonly misidentified lines<br>(See ICLAC register)	None

Animals and other research organisms

Policy information about studies involving animals; ARRIVE guidelines recommended for reporting animal research, and Sex and Gender in Research

Laboratory animals	Previously generated (Miller, P.H.. et al, Exp. Hematol., 2017) NOD.Cg-Rag1tm1Mom KitW-41J Il2rgtm1Wjl/EavJ (NRG-W41) mice (only female and 8-12 weeks old at transplant) were used for in vivo work. Female mice are used as this gives higher engraftment (Iannciello et al. Science Translational Medicine, volume 13. issue 613, (2021)). Bone marrow was isolated from the hips and hind limbs through centrifugation as described in sample preparation section.
Wild animals	No wild animals were used in the study.
Reporting on sex	Only female mice were used for all PDX experiments as engraftment is low and variable in male mice
Field-collected samples	No field collected samples were used in the study.
Ethics oversight	All animal experiments were conducted in accordance with the regulations outlined by the Animals (Scientific Procedures) Act. All experiments were conducted using personal licence number IE2DD924E and project licence PP2518370.

Note that full information on the approval of the study protocol must also be provided in the manuscript.

Clinical data

Policy information about clinical studies
All manuscripts should comply with the ICMJEguidelines for publication of clinical research and a completedCONSORT checklist must be included with all submissions.

Clinical trial registration	N/A
Study protocol	N/A
Data collection	N/A
Outcomes	N/A

Flow Cytometry

Plots

Confirm that:

☒ The axis labels state the marker and fluorochrome used (e.g. CD4-FITC).

☒ The axis scales are clearly visible. Include numbers along axes only for bottom left plot of group (a 'group' is an analysis of identical markers).

☒ All plots are contour plots with outliers or pseudocolor plots.

☒ A numerical value for number of cells or percentage (with statistics) is provided.

Methodology

Sample preparation	Cell suspensions were prepared from the bone marrow isolated from the hind limbs of NRGW41. This was done by placing inverted cut leg bones into 0.5mL Eppendorf tubes with holes at bottom. These in turn were placed within 1.5mL Eppendorf tubes containing PBS, centrifuged (12,000 RCF, 20 seconds). Cells were resuspended in 2% FBS/PBS solution and stained with monoclonal antibodies to mouse CD45, human CD45, CD34 and CD38. Stained cells were analysed on FACSVerseTM Flow Cytometer (BD Biosciences) or sorted using purity sort mode. lowest flow-rate (1), on a BD FACSAria Fusion (BD Biosciences, chilled to 4C).Data represented as percentage of whole bone marrow and absolute cell number calculated from whole bone marrow counts.
Instrument	FACSVerseTM Flow Cytometer (BD Biosciences) or BD FACSAria Fusion (BD Biosciences)
Software	BD FACSuite (BD Biosciences, FACSVerseTM Flow Cytometer, BD FACSuite version 1.0.6.5320) or FACSDiva (BD Biosciences, FACSAria Fusion, BD FACSDiva Software v8.0.1). Flow cytometry data was analyzed using FlowJo version 10
Cell population abundance	CD34+CD38- and CD34+CD38+ was assessed using Fluorescence minus one controls and typically was 10-15:90-85%. Purity sort mode was used (>95% purity, routinely confirmed using manufacturing recommended beads.
Gating strategy	For all flow cytometry assays, cells were firstly gated by FSC/SSC to exclude debris. Gating strategy is shown in supplementary data file.

☒ Tick this box to confirm that a figure exemplifying the gating strategy is provided in the Supplementary Information.
